# Incidence and risk factors of postoperative delirium in children with congenital heart disease

**DOI:** 10.1371/journal.pone.0329716

**Published:** 2026-07-17

**Authors:** Ru-Gang An, Jin-Qing Tang, Jing Xu, Hao Xie, Bo-Fei Dong, Yan Wang, Zheng-Quan Tan, Ming-Qiang Zhou

**Affiliations:** 1 Department of Anesthesiology, The First People’s Hospital of Zunyi (The Third Affiliated Hospital of Zunyi Medical University), Zunyi, Guizhou Province, China; 2 Department of Neonatology, The First People’s Hospital of Zunyi (The Third Affiliated Hospital of Zunyi Medical University), Zunyi, Guizhou Province, China; 3 Division of Orthopaedic Surgery, Department of Orthopaedics, Nanfang Hospital, Southern Medical University, Guangzhou, Guangdong, China; 4 The Second School of Clinical Medicine, Southern Medical University, Guangzhou, Guangdong, China; 5 Department of Rehabilitation Medicine, Guizhou Aerospace Hospital, Zunyi, Guizhou Province, China; UKSH Campus Lübeck, GERMANY

## Abstract

**Background:**

Postoperative delirium (POD) is an important neurocognitive complication following congenital heart disease (CHD) surgery in pediatric patients. While recognized for its association with adverse clinical outcomes, comprehensive population-based studies examining POD incidence and risk factors in this vulnerable population remain scarce.

**Methods:**

Using the Nationwide Inpatient Sample (NIS) database (2010–2019), a retrospective study was performed of pediatric patients (<18 years) undergoing CHD-related surgeries. We evaluated demographics (race, sex, age), hospital characteristics (region of the hospital, type of admission, bed size of hospital, teaching status of hospital, type of insurance, location of hospital), mortality, length of stay (LOS), total charges, perioperative complications, and comorbidities.

**Results:**

Among 187,272 CHD surgery patients identified, POD was diagnosed in 3,857 (incidence 2.1%). Children with POD exhibited higher comorbidity burdens, increased total medical costs, prolonged LOS and increased rates of in-hospital mortality (P < 0.001). Independent predictors of POD included coagulopathy, fluid and electrolyte disorders, and preexisting paralysis. Additionally, POD was associated with in-hospital complications, including prolonged mechanical ventilation, acute renal failure, acute myocardial infarction, pneumonia, and respiratory failure.

**Conclusions:**

Although the incidence of POD following CHD surgery in children is relatively low, investigating its predisposing factors is clinically valuable for optimizing patient management and improving outcomes.

## Introduction

Congenital heart disease (CHD) represents the most prevalent birth defect globally, affecting approximately 1.6% of live births [1]. Although advances in medical technology and surgical techniques have dramatically improved survival rates for children with CHD, making cardiac surgery a critical life-saving intervention, postoperative complications remain a significant concern [2,3]. Currently, approximately 40,000 pediatric cardiac surgeries are performed annually in the United States alone, with this number projected to increase due to enhanced diagnostic capabilities and improved healthcare accessibility [4].

Postoperative delirium (POD) is one of the most common and serious complications following CHD surgery in children [5,6]. Postoperative delirium is an acute neuropsychiatric syndrome characterized by alterations in attention, awareness, and cognition, typically emerging within hours to days after surgery [7]. The reported incidence varies substantially, ranging from 10% to 80%, depending on diagnostic criteria and patient populations [8,9]. Evidence demonstrates that pediatric CHD patients developing POD experience significantly worse clinical outcomes, including longer periods of mechanical ventilation, extended durations of hospital and ICU admissions, and heightened healthcare expenses, and long-term cognitive impairment [10–12]. Beyond financial burdens, POD has been shown to adversely affect patients, families, and healthcare systems, leading to higher mortality rates, progressive functional impairment, and reduced quality of life [13,14].

The etiology of POD in pediatric CHD patients is multifactorial. Numerous risk factors have been recognized, such as the patient’s age, the severity of the CHD, and any cognitive impairments present before surgery [15–17]. Additionally, factors such as cyanotic heart disease, developmental delays, and postoperative electrolyte disturbances have been linked to an increased risk of delirium [9,18]. Although research on POD in pediatric patients undergoing CHD surgery is increasing, many current studies are limited by small sample sizes, single-center designs, or inconsistent diagnostic criteria.

To address this gap, we analyzed a national estimate of 187,272 pediatric CHD surgery cases from the 2010–2019 National Inpatient Sample (NIS) database. This study aims to elucidate the epidemiology of POD, identify key risk factors, and inform targeted prevention strategies to improve clinical outcomes in this vulnerable population.

## Materials and methods

### Data source

This study employed data from the NIS, a principal database within the Healthcare Cost and Utilization Project (HCUP) supported by the Agency for Healthcare Research and Quality (AHRQ). As the largest all-payer database of hospital admissions in the United States, the NIS provides a comprehensive and stratified sample of over 1,000 hospitals, representing approximately 20% of annual hospitalizations nationwide. This database includes detailed information on patient demographics, hospital characteristics, length of stay (LOS), total hospitalization charges, payer types, in-hospital mortality, and diagnostic and procedural codes based on the International Classification of Diseases, Ninth and tenth revisions, Clinical Modification (IICD-9-CM and ICD-10-CM). Discharge weights (DISCWTs) are supplied for each admission and used toproduce national estimates. Postoperative delirium was identified using ICD-9-CM and ICD-10-CM codes. The use of ICD-based definitions for delirium in pediatric administrative data has been validated, demonstrating high specificity for detecting true cases, although sensitivity is comparatively lower and may result in underascertainment of milder or transient episodes [19,20]. For this retrospective analysis, de-identified administrative data were obtained from HCUP-NIS under the HCUP Data Use Agreement. The NIS database’s robust sampling methodology and extensive coverage make it a reliable source for studying the incidence and risk factors of POD in children with CHD.

### Data access and ethics statement

This cross-sectional nationwide study analyzed fully de-identified patient data from the 2010–2019 US National Inpatient Sample (NIS) database (HCUP). Research data access occurred between 01/09/2024 and 15/12/2024 with no accessible protected health information at any phase. All NIS records were pre-anonymized prior to release with no identifiable personal information retained. The Institutional Review Board of The First People’s Hospital of Zunyi (The Third Affiliated Hospital of Zunyi Medical University) granted exemption from full ethical review and waived informed consent requirements, as researchers accessed only de-identified data in compliance with HCUP Data Use Agreement protocols.

### Data collection

**Fig 1:**
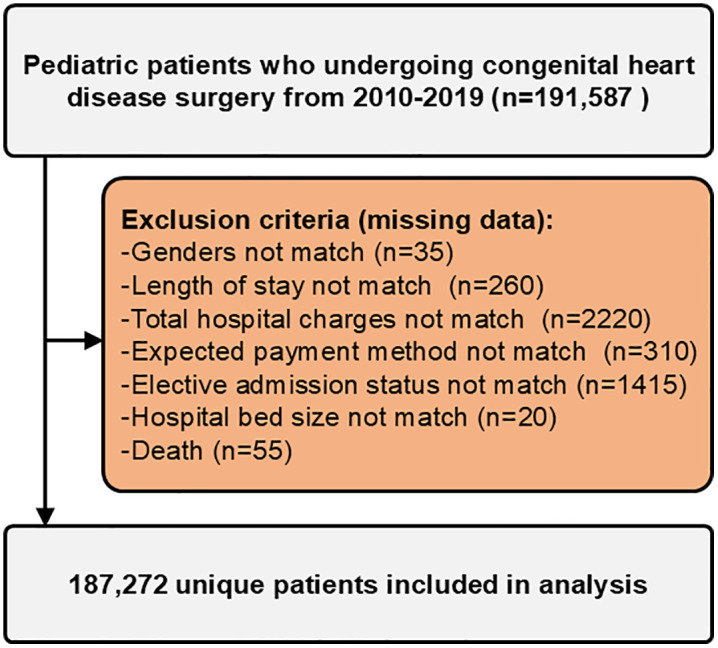
Exclusion process for pediatric patients undergoing congenital heart disease with postoperative delirium.

The study analyzed 2010−2019 NIS data, and the ICD-9-CM and ICD-10-CM was utilized to identify patients who had surgery for CHD and to assess the incidence of POD. Study inclusion criteria encompassed patients under 18 years of age who underwent surgical intervention for CHD. Postoperative delirium was diagnosed using particular ICD-9-CM and ICD-10-CM diagnostic codes [21] (Supplemental Table 1). Initially, 191,587 cases were identified. After excluding patients with missing data for key variables, such as gender, total hospital charges, length of stay, expected payment method, elective admission status, hospital bed size and death, the final analysis comprised 187,272 cases (Fig 1).

**Table 1:** Variables used in binary logistic regression analysis.

Variables Categories	Specific Variables
Patient demographics	Age (0–2years/3–5years/6–12years/13–17years/), sex (male and female), race (White, Black, Hispanic, Asian or Pacific Islander, Native American and Other)
Hospital characteristics	Type of admission (non-elective, elective), bed size of hospital (small, medium, large), teaching status of hospital (nonteaching, teaching), location of hospital (rural, urban), type of insurance (Medicare and Medicaid, private insurance, self-pay, no charge, other), region of the hospital (northeast, Midwest or north central, south, west)
Comorbidities	Deficiency anemia, chronic blood loss anemia, congestive heart failure, chronic pulmonary disease, coagulopathy, depression, diabetes (with chronic complications), hypertension, hypothyroidism, liver disease, lymphoma, fluid and electrolyte disorders, metastatic cancer, obesity, paralysis, peripheral vascular disorders, psychoses, pulmonary circulation disorders, renal failure, valvular disease and weight loss

Patient demographics (including race, sex, and age), hospital characteristics (region of the hospital, type of admission, bed size of hospital, teaching status of hospital, location of hospital, type of insurance), and outcome measures such as length of stay (LOS), and total hospital charges (TOTCHG) and in-hospital mortality were extracted from the database. Additionally, information on preoperative comorbidities and perioperative complications was collected using ICD-9-CM and ICD-10-CM diagnostic codes (Table 1). Perioperative complications included: pneumonia, blood transfusion, continuous invasive mechanical ventilation, urinary tract infection, acute renal failure, wound dehiscence/non-healing, acute myocardial infarction, arrhythmia, postoperative shock, and respiratory failure.

### Data analysis

Statistical analysis was performed using IBM SPSS Statistics 29.0. Descriptive statistics were used to summarize patient demographics, hospital characteristics, and clinical outcomes. Continuous variables, such as age and length of stay (LOS), were analyzed using independent t-tests, while categorical variables, such as gender, race, and insurance type, were compared using chi-square tests. Univariate analyses evaluated potential associations between POD and demographic/clinical variables. Results are presented as adjusted odds ratios (OR) with corresponding 95% confidence intervals (CI). Due to the occurrence of extreme odds ratios and the potential for sparse data bias, we performed a sensitivity analysis using Firth’s penalized-likelihood logistic regression. Given the substantial cohort size, a conservative significance threshold (α = 0.001) was adopted to minimize Type I error risk. All tests were two-tailed, and missing data were addressed through complete case analysis to maintain methodological rigor.

Independent predictors were discerned by means of stepwise binary logistic regression, incorporating patient demographics, hospital characteristics, and key proxies for surgical complexity and disease severity (including comorbidity count, specific high-risk comorbidities, and elective admission status).

## Results

### The incidence of postoperative delirium in patients with CHD surgery

**Fig 2:**
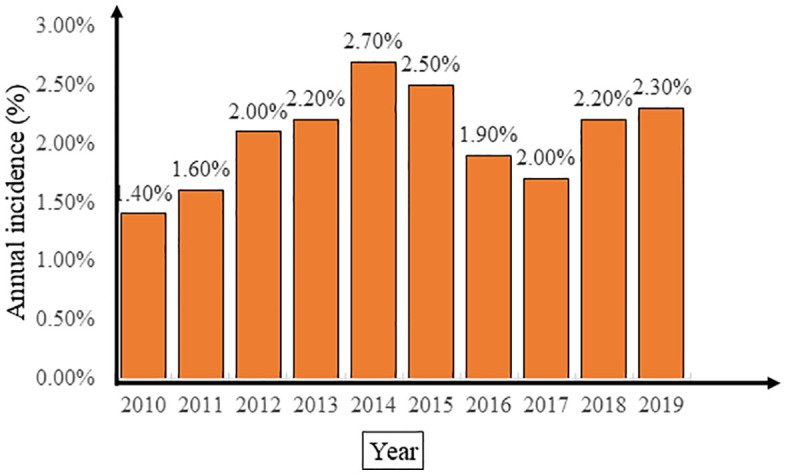
Annual Incidence of Postoperative Delirium in pediatric Patients undergoing congenital heart disease.

An estimated 187,272 children underwent CHD surgery nationally from 2010 to 2019, of whom 3,857 (2.1%) were diagnosed with POD. The annual incidence of postoperative delirium exhibited slight fluctuations over the study period, ranging from 1.4% in 2010 to 2.7% in 2014, with an overall stable trend (Fig 2). A detailed year-by-year analysis revealed that the highest incidence occurred in 2014 (2.7%, n = 495), followed by 2015 (2.5%, n = 480) and 2019 (2.3%, n = 435). In contrast, the lowest rates were observed in 2010 (1.4%, n = 257) and 2011 (1.6%, n = 230). Notably, no consistent upward or downward trajectory was observed across the decade, suggesting that postoperative delirium incidence remained relatively stable in this population (Fig 2).

### Hospital characteristics and patient demographics in two surgical groups

**Fig 3:**
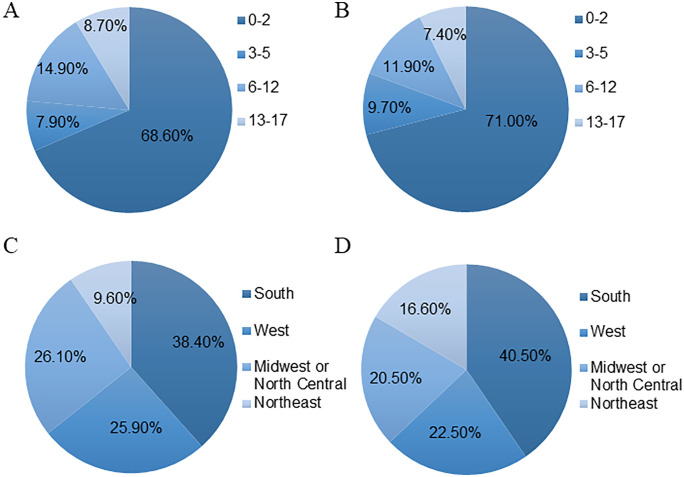
Patient demographics between the two surgical groups. A: Age distribution analysis of postoperative delirium patients. B: Analysis of age distribution of patients without postoperative delirium. C: Hospital Region of patients with postoperative delirium. D: Hospital Region of patients without postoperative delirium.

**Fig 4:**
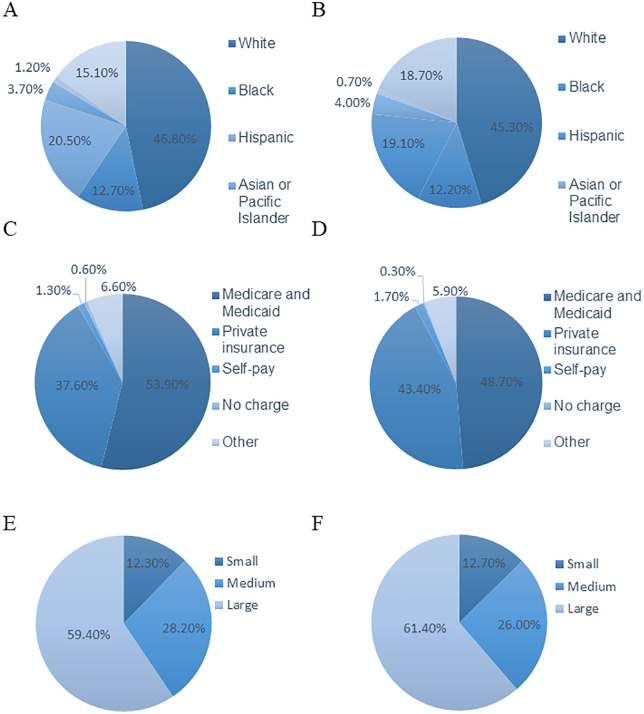
Racial distribution and incidence of Postoperative Complications Related to Postoperative Delirium. A Racial distribution analysis of delirium patients. B Racial distribution analysis of non-delirium patients. C Analysis of Insurance Types for Patients with Delirium. D Analysis of Insurance Types for Non-delirium Patients. E Analysis of the number of hospital beds for patients with delirium. F Analysis of the number of hospital beds for non-delirium patients.

Significant differences were observed in age-related characteristics between POD and without POD in children undergoing congenital heart surgery. Patients with POD were significantly older (median age: 0.0 [0.0–5.0] years vs 0.0 [0.0–3.0] years, P < 0.001) (Table 2). Age distribution analysis revealed markedly different patterns, with the POD group having lower proportions of infants (0–2 years: 68.6% vs 71.0%) but higher proportions of older children (6–12 years: 14.9% vs 11.9%; 13–17 years: 8.7% vs 7.4%, P < 0.001) (Table 2 and Fig 3A & B).The elective admission rate was significantly lower in the POD group compared to non-POD patients (44.1% vs 61.7%, P < 0.001) (Table 2). Regional variations were also significant, with higher POD proportions in the Midwest/North Central (26.1% vs 20.5%) and West (25.9% vs 22.5%) regions (P < 0.001) (Table 2 and Fig 3C & D). However, there were no statistical differences in variables such as race, gender, insurance type, hospital type, region of hospital, bed size of hospital, etc. (Table 2 and Fig 4 A & B & C and D).

**Table 2:** Patient characteristics and outcomes after congenital heart disease surgery (2010-2019).

Characteristics	POD	No POD	P
Total (n = count)	3,857	183,415	
Total incidence (%)	2.1		
Age (median, years)	0.0 (0.0,5.0)	0.0 (0.0,3.0)	< 0.001
Age group (%)			
0-2	68.6	71.0	< 0.001
3-5	7.9	9.7	
6-12	14.9	11.9	
13-17	8.7	7.4	
Gender (%)			
Male	59.4	54.8	0.040
Female	40.5	45.2	
Race (%)			
White	46.8	45.3	0.026
Black	12.7	12.2	
Hispanic	20.5	19.1	
Asian or Pacific Islander	3.7	4.0	
Native American	1.2	0.7	
Other	15.1	18.7	
Number of Comorbidity (%)			
0	3.8	39.5	< 0.001
1	19.0	32.4	
2	27.4	16.4	
≥3	49.8	11.7	
LOS (median, d)	36 (17-73)	8(4-22)	< 0.001
TOTCHG (median, $)	754,512.32 (316,876.00−1,583,205.00)	187,173.00 (11,875.00−398,401.00)	< 0.001
Type of insure (%)			
Medicare and Medicaid	53.9	48.7	0.007
Private insurance	37.6	43.4	
Self-pay	1.3	1.7	
No charge	0.6	0.3	
Other	6.6	5.9	
Bed size of hospital (%)			
Small	12.3	12.7	0.006
Medium	28.2	26.0	
Large	59.4	61.4	
Elective admission (%)	44.1	61.7	< 0.001
Type of hospital (teaching %)	99.5	99.0	0.002
Location of hospital (urban, %)	99.9	99.8	0.693
Region of hospital (%)			
Northeast	9.6	16.6	
Midwest or North Central	26.1	20.5	< 0.001
South	38.4	40.5	
West	25.9	22.5	
Died during hospitalization	13.1%	3.1%	< 0.001

### Adverse outcomes of postoperative delirium after CHD in Children

Congenital heart disease children with POD demonstrated a significantly greater comorbidity burden, with 49.80% presenting ≥3 coexisting conditions compared to 11.70% in non-POD cases (P < 0.001) (Table 2). Compared with the non‑delirium group, the mortality rate in the delirium group increased approximately fourfold (13.1% vs. 3.1%; P < 0.001) (Table 2). Hospitalization duration showed marked prolongation, with median length of stay (LOS) extending fourfold in POD patients (36 days vs. 8 days; P < 0.001) (Table 2). This clinical status was associated with substantial economic consequences, as the total charges of hospitalization increased by $567,339.32 ($754,512.32 vs. $187,173.00, P < 0.001) (Table 2).

### Risk Factors of POD Following Surgery for Congenital Heart Disease in Children

Logistic regression analysis identified several factors significantly associated with POD in pediatric CHD surgical patients. Older age groups were associated with increased POD risk, with children aged 6–12 years (OR = 1.827; 95% CI: 1.659–2.012) and 13–17 years (OR = 1.565; 95% CI: 1.391–1.761) showing the strongest effects (Table 3). Other factors significantly associated with POD included private insurance (OR = 0.803; 95% CI: 0.748–0.863), teaching hospital status (OR = 2.584; 95% CI: 1.549–4.311), and hospital region (Midwest/North Central: OR = 2.171; 95% CI: 1.920–2.454; South: OR = 1.520; 95% CI: 1.353–1.709; West: OR = 1.923; 95% CI: 1.699–2.176) (Table 3). Among preoperative comorbidities, coagulopathy (OR = 2.419; 95% CI: 2.228–2.626), fluid and electrolyte disorders (OR = 2.570; 95% CI: 2.402–2.749), and preexisting paralysis (OR = 4.686; 95% CI: 3.980–5.519) were independently associated with POD (all P < 0.001; Table 4). Absolute risk differences for predictors in the final multivariable model were also calculated (sTable 12). Collinearity diagnostics revealed variance inflation factors (VIFs) below 2.0 for all predictors in the final model (sTable 11), indicating no substantial multicollinearity. The overall event-per-variable ratio for the final multivariable model was approximately 22:1, which exceeded the recommended minimum threshold of 10:1 and further validated model stability. Additionally, Firth penalized logistic regression produced consistent estimates, solidifying the overall robustness of our final model (sTable 2 and 3).

**Table 3:** Risk factors associated with postoperative delirium following Surgery for Congenital Heart Disease in Children.

Variable	Multivariate Logistic Regression		
OR	95% CI	P
Age group (%)			
0-2	Ref	——	——
3-5	1.231	1.087-1.395	0.001
6-12	1.827	1.659-2.012	< 0.001
13-17	1.565	1.391-1.761	< 0.001
Female	0.855	0.733-0.996	0.044
Race			
White	Ref	——	——
Black	0.970	0.874-1.077	0.569
Hispanic	0.976	0.892-1.067	0.590
Asian or Pacific Islander	0.919	0.771-1.095	0.414
Native American	1.497	1.103-2.033	0.010
Other	0.813	0.738-0.895	< 0.001
Type of insurance			
Medicare/ Medicaid	Ref	——	——
Private insurance	0.803	0.748-0.863	< 0.001
Self-pay	0.740	0.558-0.983	0.038
No charge	2.131	1.391-3.262	0.001
Other	0.987	0.863-1.129	0.850
Bed size of hospital			
Small	Ref	——	——
Medium	1.122	1.001-1.257	0.047
Large	0.989	0.890-1.09	0.832
Elective admission	0.431	0.402-0.462	< 0.001
Teaching hospital	2.584	1.549-4.311	< 0.001
Urban hospital	0.352	0.126-0.980	0.046
Region of hospital			
Northeast	Ref	——	——
Midwest or North Central	2.171	1.920-2.454	< 0.001
South	1.520	1.353-1.709	< 0.001
West	1.923	1.699-2.176	< 0.001

**Table 4:** Relationship between postoperative Delirium and preoperative comorbidities.

Comorbidities	Univariate Analysis			Multivariate Logistic Regression		
No POD	POD	P	OR	95% CI	P
Preoperative comorbidities						
Deficiency anemia	6,796 (3.7%)	277(7.2%)	< 0.001	1.492	1.309-1.699	0.001
Chronic blood loss anemia	667 (0.4%)	25(0.6%)	0.006	1.045	0.696-1.570	0.831
Congestive heart failure	20,575 (11.2%)	930 (24.1%)	< 0.001	1.626	1.493-1.772	0.001
Chronic pulmonary disease	7,278 (4.0%)	215 (5.6%)	< 0.001	1.287	1.115-1.486	0.001
Coagulopathy	12,966 (7.1%)	888 (23.0%)	< 0.001	2.419	2.228−2.626	< 0.001
Depression	598 (0.3%)	35 (0.9%)	0.001	1.752	1.219-2.518	0.002
Diabetes with chronic complications	95 (0.1%)	5 (0.1%)	0.086	0.920	0.360-2.355	0.862
Hypertension	18,215 (9.9%)	569 (14.8%)	< 0.001	1.188	1.083-1.306	< 0.001
Hypothyroidism	4,516 (2.5%)	80 (2.1%)	0.123	0.607	0.483-0.762	< 0.001
Liver disease	1,744 (1.0%)	110 (2.9%)	< 0.001	1.445	1.174-1.779	< 0.001
Lymphoma	40 (0.0%)	5 (0.1%)	< 0.001	3.350	1.248-8.994	0.016
Fluid and electrolyte disorders	47,702 (26.0%)	2,096 (54.3%)	< 0.001	2.570	2.402-2.749	< 0.001
Metastatic cancer	136 (0.1%)	10 (0.3%)	< 0.001	2.623	1.332-5.167	0.005
Obesity	1,480 (0.8%)	30 (0.8%)	0.912	0.691	0.471-1.013	0.058
Paralysis	1,426 (0.8%)	190 (4.9%)	< 0.001	4.686	3.980-5.519	< 0.001
Peripheral vascular disorders	6,252 (3.4%)	315 (8.2%)	< 0.001	1.880	1.663-2.125	< 0.001
Psychoses	199 (0.1%)	25 (0.6%)	< 0.001	4.656	2.998-7.232	< 0.001
Pulmonary circulation disorders	8,467 (4.6%)	440 (11.4%)	< 0.001	1.490	1.334-1.664	< 0.001
Renal failure	856 (0.5%)	101 (2.6%)	< 0.001	2.964	2.372-3.703	< 0.001
Valvular disease	40,717 (22.2%)	1,085 (28.1%)	< 0.001	1.025	0.949-1.107	0.534
Weight loss	8,150 (4.4%)	445 (11.5%)	< 0.001	1.643	1.474-1.831	< 0.001

### Risk factors associated with postoperative delirium after CHD surgery

Postoperative delirium occurred more frequently in pediatric CHD patients with acute myocardial infarction, acute renal failure, pneumonia, urinary tract infection, continuous mechanical ventilation, arrhythmia, wound dehiscence/non-healing, postoperative shock and respiratory failure (*P* < 0.001) (Table 5[href:https://pmc.ncbi.nlm.nih.gov/articles/PMC10953239/]). Multivariable regression analysis revealed a significant association between POD and acute renal failure (OR = 3.325; 95% CI: 3.076–3.595; P < 0.001), acute myocardial infarction (OR = 1.718; 95% CI: 1.528–1.931; P < 0.001), pneumonia (OR = 3.448; 95% CI: 3.138–3.790; P < 0.001), continuous mechanical ventilation (OR = 1.801; 95% CI: 1.676–1.936; P < 0.001), urinary tract infection (OR = 1.601; 95% CI: 1.394–1.838; P < 0.001), wound dehiscence (OR = 1.553; 95% CI: 1.320–1.826; P < 0.001), arrhythmia (OR = 1.292; 95% CI: 1.142–1.461; P < 0.001), postoperative shock (OR = 1.914; 95% CI: 1.619–2.263; P < 0.001), and respiratory failure (OR = 2.024; 95% CI: 1.881–2.177; P < 0.001) (Table 5).

**Table 5:** Relationship between postoperative delirium and postoperative complications.

Complication	Univariate Analysis			Multivariate Logistic Regression		
No POD	POD	P	OR	95% CI	P
Medical complications						
Acute renal failure	12,818 (7.0%)	1,064 (27.6%)	< 0.001	3.325	3.076-3.595	< 0.001
Acute myocardial infarction	7,720 (4.2%)	370(9.6%)	< 0.001	1.718	1.528-1.931	< 0.001
Pneumonia	5,998 (3.3%)	639 (16.6%)	< 0.001	3.448	3.138-3.790	< 0.001
Blood transfusion	53,037 (28.9%)	1,109(28.8%)	0.839	0.867	0.806-0.932	< 0.001
Continuous mechanical ventilation	31,372(17.1%)	1,342(34.8%)	< 0.001	1.801	1.676-1.936	< 0.001
Urinary tract infection	3,842 (2.1%)	261 (6.8%)	< 0.001	1.601	1.394-1.838	< 0.001
Wound dehiscence	3,441 (1.9%)	174 (4.5%)	< 0.001	1.553	1.320-1.826	< 0.001
Arrhythmia	9,450 (5.2%)	315 (8.2%)	< 0.001	1.292	1.142-1.461	< 0.001
Postoperative shock	2,297 (1.3%)	175 (4.5%)	< 0.001	1.914	1.619-2.263	< 0.001
Respiratory failure	25,362 (13.8%)	1,243 (32.2%)	< 0.001	2.024	1.881-2.177	< 0.001

## Discussion

Postoperative delirium represents a significant complication in pediatric CHD surgery that substantially impacts recovery outcomes. This large-scale health economic analysis characterizes POD epidemiology across pediatric CHD surgical populations. Our study identified an overall POD incidence of 2.1% between 2010–2019, demonstrating temporal stability. This finding contrasts markedly with Mao et al.‘s single-center study reporting 25.4% POD incidence following pediatric CHD surgery [15]. This disparity likely reflects fundamental methodological differences between multicenter administrative data analysis and prospective clinical assessments. The ICD‑based diagnostic criteria are more stringent than DSM psychiatric classifications, leading to lower case identification rates [22,23]. Additionally, several inherent limitations of the NIS database contribute to potential underestimation of delirium incidence: the database captures only in‑hospital delirium cases and has high specificity but low sensitivity, resulting in systematic underdetection [22–24]. Moreover, ICD‑9‑CM and ICD‑10‑CM codes cannot adequately document key delirium manifestations such as disorganized thinking, altered consciousness, cognitive deficits, and perceptual disturbances [14,25]. These methodological constraints suggest that our reported incidence is a conservative estimate of true POD prevalence in this vulnerable population.

Regarding patient demographics, our analysis identified older age as a significant risk factor for POD following CHD surgery in the pediatric population. Children aged 6 years and above had significantly higher odds of POD than the youngest age group. This finding contradicts previous reports associating younger age with higher delirium risk [26,27]. This discrepancy primarily reflects differential detection bias: hypoactive delirium in infants is less frequently coded than the hyperactive phenotype in school-aged children, attenuating risk estimates in the youngest group [28,29]. Methodological differences (single-center tertiary studies skewed toward neonates vs. nationwide representative sampling of school-aged children undergoing elective procedures) may also contribute.

Variation in POD incidence across hospitals (by region, teaching status, insurance, and admission type) may partly reflect differences in patient characteristics and care processes. Teaching hospital status was independently associated with POD (OR = 2.584), which may reflect greater diagnostic vigilance or higher patient complexity at academic centers. Regionally, compared with the Northeast, the Midwest/North Central (OR = 2.171), South (OR = 1.520), and West (OR = 1.923) all had higher POD odds. Private insurance was protective (OR = 0.803), possibly indicating better baseline health or access to care [30,31]. Interestingly, our regression analysis identified elective admission as a significant protective factor against POD, corroborating existing literature that demonstrates reduced delirium risk in electively admitted patients [32,33]. This protective effect may be attributed to patients’ more stable baseline health status and the opportunity for comprehensive preoperative optimization in elective cases. The observed variations in POD incidence across hospitals are largely attributable to differences in patient case‑mix and care processes rather than fixed institutional or regional characteristics. However, we cannot rule out the influence of differential coding practices or documentation intensity. For instance, regions with lower reported POD incidence may have lower coding propensity for delirium, consistent with the known low sensitivity of ICD-based identification [34,35]. These interpretations should be viewed cautiously given the limitations of administrative data (sTable 4–10).

Our analysis further revealed that preexisting comorbidities substantially increased the risk of postoperative delirium in this population. Comorbid conditions may predispose pediatric patients to heightened physiological stress and chronic inflammation, which when combined with the surgical stress-induced inflammatory response, can potentially trigger neuroinflammation and precipitate postoperative delirium [36–38]. Consistent with previous literature [14,39], our investigation found that POD is associated with coagulopathy, fluid and electrolyte disorders, and preexisting paralysis. Beyond these risk factors, POD itself was associated with adverse outcomes. Studies have demonstrated that POD is significantly associated with prolonged hospitalization, likely attributable to the extended monitoring and specialized care required for delirious patients [8,40]. Moreover, POD increases healthcare costs through multiple mechanisms, including heightened consumption of medical resources (e.g., frequent monitoring, pharmacological therapies, and nursing interventions) and the necessity of additional treatments for delirium-associated complications such as infections or respiratory failure [41,42]. Most concerningly, POD was correlated with elevated in‑hospital mortality, potentially mediated by delirium-induced life-threatening complications (e.g., infections, organ dysfunction) or heightened exposure to iatrogenic risks during prolonged hospitalization [5,43]. Nevertheless, due to the observational nature of our study, this association should not be interpreted as causal; POD may reflect greater baseline disease severity or physiological instability rather than directly causing death.

Our retrospective analysis demonstrates significant associations between POD and a wide range of perioperative complications. After multivariable adjustment, acute renal failure (OR = 3.325), acute myocardial infarction (OR = 1.718), pneumonia (OR = 3.448), continuous mechanical ventilation (OR = 1.801), urinary tract infection (OR = 1.601), wound dehiscence (OR = 1.553), arrhythmia (OR = 1.292), postoperative shock (OR = 1.914), and respiratory failure (OR = 2.024) were all significantly associated with POD (all P < 0.001). While these associations are strong, the observational nature of the study precludes definitive causal attribution. Postoperative delirium may both predispose to complications (e.g., through aspiration, immobility, or poor cooperation) and be a marker of greater illness severity. Prospective studies are needed to disentangle directionality.

This study has the following limitations. First, as a retrospective analysis of an administrative database, the observational design precludes causal inference. While we identified significant associations, temporal ambiguity and unmeasured confounding mean our findings should be interpreted as correlations rather than causal effects. Second, delirium identification relied on ICD‑9‑CM and ICD‑10‑CM codes, which have high specificity but low sensitivity. This leads to systematic underdetection, especially of hypoactive delirium in infants. Such non‑differential misclassification would bias effect estimates toward the null, so our findings are likely conservative. Coding practices also vary across hospitals and over time, for example around the ICD‑9 to ICD‑10 transition in October 2015, which could introduce differential misclassification. Third, the NIS captures only in‑hospital events, so we cannot assess delayed‑onset POD or post‑discharge complications. Fourth, the database lacks direct measures of surgical complexity, cardiopulmonary bypass duration, intraoperative physiologic data, and key perioperative factors such as anesthesia type, sedative exposure, and cyanotic status. We used preoperative comorbidities and severe postoperative complications as proxies, but residual confounding remains likely. Fifth, approximately 2% of patients were excluded due to missing variables, a small proportion unlikely to substantially bias results. Prospective multicenter studies using standardized, age‑appropriate delirium screening tools, such as the CAPD for younger children and the pCAM‑ICU for older children, along with detailed perioperative data, are needed to validate our findings.

## Conclusions

The incidence of POD after pediatric CHD surgery was 2.1% between 2010 and 2019. Older age (especially 6‑17 years), coagulopathy, fluid and electrolyte disorders, preexisting paralysis, teaching hospital status, and geographic region (Midwest, South, West) were identified as risk factors, whereas elective admission and private insurance were protective. Postoperative delirium was associated with multiple postoperative complications, including acute renal failure, myocardial infarction, pneumonia, mechanical ventilation, urinary tract infection, wound dehiscence, arrhythmia, shock, and respiratory failure, as well as a more than four‑fold higher in‑hospital mortality. Paralysis was newly recognized as a pediatric‑specific risk factor, extending adult neuroinflammatory models to children. These findings highlight the importance of preoperative risk stratification, vigilant delirium screening, and early intervention to improve outcomes in this vulnerable population.

## Supporting information

S1 File Supplemental Table. Includes sTable 1–sTable12.
(DOCX)
