# Supplementary material for: Incidence and risk factors of postoperative delirium in children with congenital heart disease
Source: PLoS One. 2026 Jul 17;21(7):e0329716. doi: 10.1371/journal.pone.0329716 (PMC13378989; doi:10.1371/journal.pone.0329716)
Supplement: S1 File — Includes sTable 1–sTable12. (DOCX) [file pone.0329716.s001.docx]

sTable 1. ICD‑9‑CM and ICD‑10‑CM codes used to identify POD, with frequency of dementia‑related codes in the POD cohort

| Diagnosis Codes | | | | | | |
| --- | --- | --- | --- | --- | --- | --- |
| ICD‑9‑CM | Frequency | ICD 9 CM | | Frequency | ICD 9 CM | Frequency |
| 293 | 228 | 292.81 | | 0 | 292.12 | 30 |
| 293.1 | 0 | 290.11 | | 0 | 292.2 | 0 |
| 292.81 | 70 | 290.3 | | 0 | 780.02 | 30 |
| 290.11 | 0 | 290.41 | | 0 | 290.2 | 0 |
| 290.3 | 0 | 291 | | 0 | 290.42 | 0 |
| 290.41 | 0 | 293.9 | | 0 | 290.8 | 0 |
| 291 | 0 | 780.09 | | 0 | 290.9 | 0 |
| 293.9 | 84 | 293.81 | | 0 | 292 | 980 |
| 780.09 | 155 | 293.82 | | 0 | 292.82 | 0 |
| 293.81 | 0 | 293.83 | | 0 | 348.3 | 690 |
| 293.82 | 0 | 293.84 | | 0 | 348.31 | 31 |
| 293.83 | 0 | 293.89 | | 0 | 348.39 | 287 |
| 293.84 | 10 | 290.12 | | 0 | 349.82 | 44 |
| 293.89 | 5 | 290.13 | | 0 | 780.97 | 169 |
| 293 | 0 | 290.43 | | 0 |  | |
| 293.1 | 0 | 292.11 | | 0 |  | |
| ICD-10 version | | | | | | |
| ICD‑10‑CM | Frequency | ICD 10 CM | Frequency | | ICD 10 CM | Frequency |
| F05 | 405 | F14921 | 0 | | G0431 | 0 |
| F10121 | 0 | F15121 | 0 | | G0432 | 0 |
| F10221 | 0 | F15221 | 0 | | G0439 | 0 |
| F10231 | 0 | F15921 | 0 | | G92 | 60 |
| F10921 | 0 | F16121 | 0 | | G9340 | 585 |
| F11121 | 0 | F16221 | 0 | | G9341 | 40 |
| F11221 | 0 | F16921 | 0 | | G9349 | 200 |
| F11921 | 0 | F18121 | 0 | | I673 | 0 |
| F12121 | 0 | F18221 | 0 | | I674 | 5 |
| F12221 | 0 | F18921 | 0 | | I6783 | 25 |
| F12921 | 0 | F19121 | 0 | | J1081 | 0 |
| F13121 | 0 | F19221 | 5 | | J1181 | 0 |
| F13221 | 0 | F19231 | 0 | | P9160 | 240 |
| F13231 | 50 | F19921 | 15 | | P9161 | 15 |
| F13921 | 5 | F19931 | 0 | | P9162 | 40 |
| F13931 | 0 | A812 | 0 | | P9163 | 65 |
| F14121 | 0 | E512 | 0 | |  | |
| F14221 | 0 | G0430 | 0 | |  | |

| **sTable 2 Risk factors associated with postoperative delirium following surgery for congenital heart disease in children: results from Firth penalized logistic regression** | | | |
| --- | --- | --- | --- |
| Comorbidities | | Firth Logistic Regression | |
|  |  | OR | 95% CI |
| Preoperative comorbidities | |  |  |
|  | Deficiency anemia | 1.478 | 1.094-1.958 |
|  | Chronic blood loss anemia | 1.123 | 0.412-2.460 |
|  | Congestive heart failure | 1.626 | 1.341-1.964 |
|  | Chronic pulmonary disease | 1.294 | 0.928-1.760 |
|  | Coagulopathy | 2.419 | 2.009-2.899 |
|  | Depression | 1.900 | 0.778-3.822 |
|  | Diabetes with chronic complications | 1.279 | 0.133-5.759 |
|  | Hypertension | 1.191 | 0.962-1.463 |
|  | Hypothyroidism | 0.625 | 0.364-0.999 |
|  | Liver disease | 1.462 | 0.899-2.265 |
|  | Lymphoma | 4.475 | 0.436-24.168 |
|  | Fluid and electrolyte disorders | 2.561 | 2.205-2.977 |
|  | Metastatic cancer | 3.171 | 0.607-10.603 |
|  | Obesity | 0.756 | 0.299-1.586 |
|  | Paralysis | 4.693 | 3.222-6.664 |
|  | Peripheral vascular disorders | 1.894 | 1.429-2.467 |
|  | Psychoses | 4.995 | 1.735-11.903 |
|  | Pulmonary circulation disorders | 1.493 | 1.160-1.900 |
|  | Renal failure | 2.968 | 1.764-4.751 |
|  | Valvular disease | 1.026 | 0.862-1.217 |
|  | Weight loss | 1.647 | 1.286-2.087 |

| **sTable 3. Preoperative comorbidity distribution in children with and without postoperative delirium after congenital heart disease surgery** | | | |
| --- | --- | --- | --- |
| Comorbidities | | Univariate Analysis | |
|  |  | No POD | POD |
| Preoperative comorbidities | |  |  |
|  | Deficiency anemia | 1,370(3.7%) | 55 (7.7%) |
|  | Chronic blood loss anemia | 135 (0.4%) | 4 (0.6%) |
|  | Congestive heart failure | 4,129 (11.2%) | 178 (24.8%) |
|  | Chronic pulmonary disease | 1,462 (4.0%) | 43 (6.0%) |
|  | Coagulopathy | 2,608 (7.1%) | 173 (24.1%) |
|  | Depression | 121 (0.3%) | 6 (0.8%) |
|  | Diabetes, uncomplicated | 33(0.1%) | 1 (0.1%) |
|  | Diabetes with chronic complications | 19(0.1%) | 1(0.1%) |
|  | Hypertension | 3656 (9.9%) | 110 (15.3%) |
|  | Hypothyroidism | 905 (2.5%) | 15 (2.1%) |
|  | Liver disease | 354 (1.0%) | 17 (2.4%) |
|  | Lymphoma | 8 (0.0%) | 1 (0.1%) |
|  | Fluid and electrolyte disorders | 9,615 (26.1%) | 391 (54.5%) |
|  | Metastatic cancer | 28 (0.1%) | 1 (0.1%) |
|  | Obesity | 294 (0.8%) | 8 (1.1%) |
|  | Paralysis | 285 (0.8%) | 39 (5.4%) |
|  | Peripheral vascular disorders | 1,259 (3.4%) | 54 (7.5%) |
|  | Psychoses | 40 (0.1%) | 5 (0.7%) |
|  | Pulmonary circulation disorders | 1,703 (4.6%) | 81 (11.3%) |
|  | Renal failure | 173 (0.5%) | 19 (2.6%) |
|  | Valvular disease | 8,178 (22.2%) | 189 (26.4%) |
|  | Weight loss | 1,642 (4.5%) | 81 (11.3%) |

| **sTable 4. Univariate Analysis of Patient Characteristics and Postoperative Delirium Incidence in large Bed-Size Hospitals** | | | | | |  |
| --- | --- | --- | --- | --- | --- | --- |
| Characteristics | | POD | No POD | | P |  |
| Total (n=count) | | 2,292 | 11,2547 |  | |  |
| Total incidence (%) | | 2.0 | |  | |  |
| Age (median, years) | | 1.0 (0.0 ,7.0) | 0.0 (0.0 ,3.0) | < 0.001 | |  |
| Age group (%) | |  |  |  | |  |
|  | 0-2 | 67.8 | 71.1 | < 0.001 | |  |
|  | 3-5 | 7.4 | 9.7 |  |  |  |
|  | 6-12 | 14.5 | 11.6 |  |  |  |
|  | 13-17 | 10.2 | 7.6 |  |  |  |
| Gender (%) | |  |  |  | |  |
|  | Male | 59.2 | 54.8 | < 0.001 | |  |
|  | Female | 40.8 | 45.2 |  |  |  |
| Race (%) | |  |  |  | |  |
|  | White | 48.5 | 47.2 | < 0.001 | |  |
|  | Black | 11.4 | 13.3 |  |  |  |
|  | Hispanic | 21.0 | 20.5 |  |  |  |
|  | Asian or Pacific Islander | 3.5 | 4.0 |  |  |  |
|  | Native American | 1.7 | 0.8 |  |  |  |
|  | Other | 11.1 | 14.1 |  |  |  |
| Type of insure (%) | |  |  |  | |  |
|  | Medicare and Medicaid | 56.7 | 50.6 | < 0.001 | |  |
|  | Private insurance | 36.5 | 42.2 |  |  |  |
|  | Self-pay | 1.5 | 1.8 |  |  |  |
|  | No charge | 0.7 | 0.2 |  |  |  |
|  | Other | 4.6 | 5.2 |  |  |  |
| Elective admission (%) | | 45.1 | 61.7 | < 0.001 | |  |
| Type of hospital (teaching %) | | 99.1 | 99.6 | 0.035 | |  |
|  | |  |  |  | |  |
| Location of hospital (urban, %) | | 99.8 | 99.7 | 0.635 | |  |
| Region of hospital (%) | |  |  |  | |  |
| Northeast | | 9.3 | 17.0 |  | |  |
| Midwest or North Central | | 24.0 | 17.9 | < 0.001 | |  |
| South | | 38.7 | 39.4 |  | |  |
| West | | 28.0 | 25.8 |  | |  |
| Died during hospitalization | | 11.3% | 3.3% | < 0.001 | |  |
| Preoperative comorbidities | | | | | |  |
| Deficiency anemia (%) | | 91.0 | 96.2 | < 0.001 | |  |
| Chronic blood loss anemia (%) | | 0.4 | 0.4 | 0.561 | |  |
| Congestive heart failure (%) | | 22.5 | 10.7 | < 0.001 | |  |
| Chronic pulmonary disease (%) | | 5.0 | 4.0 | 0.010 | |  |
| Coagulopathy (%) | | 22.7 | 7.3 | < 0.001 | |  |
| Depression (%) | | 1.1 | 0.3 | < 0.001 | |  |
| Diabetes, uncomplicated (%) | | 0.0 | 0.1 | 0.133 | |  |
| Diabetes with chronic (%) complications (%) | | 0.0 | 0.1 | 0.250 | |  |
| Hypertension (%) | | 14.8 | 8.9 | < 0.001 | |  |
| Hypothyroidism (%) | | 2.2 | 2.4 | 0.531 | |  |
| Liver disease (%) | | 2.6 | 0.8 | < 0.001 | |  |
| Lymphoma (%) | | 0.2 | 0.0 | < 0.001 | |  |
| Fluid and electrolyte disorders (%) | | 56.2 | 26.7 | < 0.001 | |  |
| Metastatic cancer (%) | | 0.2 | 0.1 | 0.034 | |  |
| Obesity (%) | | 0.9 | 0.8 | 0.733 | |  |
| Paralysis (%) | | 4.8 | 0.8 | < 0.001 | |  |
| Peripheral vascular disorders (%) | | 6.1 | 3.3 | < 0.001 | |  |
| Psychoses (%) | | 0.7 | 0.1 | < 0.001 | |  |
| Pulmonary circulation disorders (%) | | 10.9 | 4.6 | < 0.001 | |  |
| Renal failure (%) | | 2.0 | 0.4 | < 0.001 | |  |
| Valvular disease (%) | | 35.5 | 21.7 | < 0.001 | |  |
| Weight loss (%) | | 9.6 | 3.8 | < 0.001 | |  |

| **sTable 5. Univariate Analysis of Patient Characteristics and Postoperative Delirium Incidence in Medium Bed-Size Hospitals** | | | | |  |
| --- | --- | --- | --- | --- | --- |
| Characteristics | | POD | No POD | P |  |
| Total (n=count) | | 754 | 33,663 |  |  |
| Total incidence (%) | | 2.2 | |  |  |
| Age (median, years) | | 0.0 (0.0 ,5.0) | 0.0 (0.0 ,3.0) | < 0.001 |  |
| Age group (%) | |  |  |  |  |
|  | 0-2 | 69.2 | 70.7 | 0.049 |  |
|  | 3-5 | 8.7 | 9.7 |  |  |
|  | 6-12 | 15.2 | 12.4 |  |  |
|  | 13-17 | 6.9 | 7.2 |  |  |
| Gender (%) | |  |  |  |  |
|  | Male | 63.4 | 55.4 | < 0.001 |  |
|  | Female | 36.6 | 44.6 |  |  |
| Race (%) | |  |  |  |  |
|  | White | 46.8 | 47.1 | 0.065 |  |
|  | Black | 10.6 | 9.9 |  |  |
|  | Hispanic | 21.5 | 19.1 |  |  |
|  | Asian or Pacific Islander | 5.1 | 4.5 |  |  |
|  | Native American | 0.5 | 0.4 |  |  |
|  | Other | 15.6 | 18.9 |  |  |
| LOS (median, d) | | 35(15-72.5) | 8(5-22) | < 0.001 |  |
| TOTCHG (median, $) | | 862,921.00  (332,444.00-1,841,250.00) | 192,457.00  (113,476.00-416,434.00) | < 0.001 |  |
| Type of insure (%) | |  |  |  |  |
|  | Medicare and Medicaid | 42.1 | 43.9 | 0.093 |  |
|  | Private insurance | 43.9 | 46.9 |  |  |
|  | Self-pay | 1.4 | 1.5 |  |  |
|  | No charge | 0.0 | 0.1 |  |  |
|  | Other | 12.7 | 7.6 |  |  |
| Elective admission (%) | | 42.6 | 59.2 | < 0.001 |  |
| Type of hospital (teaching %) | | 100.0 | 99.9 | 0.396 |  |
| Location of hospital (urban, %) | | 100.0 | 100.0 | 0.735 |  |
| Region of hospital (%) | |  |  |  |  |
| Northeast | | 13.8 | 21.5 |  |  |
| Midwest or North Central | | 14.7 | 16.6 | < 0.001 |  |
| South | | 40.2 | 39.7 |  |  |
| West | | 31.3 | 22.2 |  |  |
| Died during hospitalization | | 16.1% | 3.0% | < 0.001 |  |
| Preoperative comorbidities | | | | |  |
| Deficiency anemia (%) | | 2.3 | 3.6 | 0.021 |  |
| Chronic blood loss anemia (%) | | 0.3 | 0.5 | 0.363 |  |
| Congestive heart failure (%) | | 26.2 | 11.4 | < 0.001 |  |
| Chronic pulmonary disease (%) | | 6.0 | 3.6 | < 0.001 |  |
| Coagulopathy (%) | | 22.0 | 6.3 | < 0.001 |  |
| Depression (%) | | 0.0 | 0.3 | 0.114 |  |
| Diabetes, uncomplicated (%) | | 0.0 | 0.1 | 0.285 |  |
| Diabetes with chronic complications (%) | | 0.0 | 0.0 | 0.499 |  |
| Hypertension (%) | | 13.8 | 11.7 | 0.037 |  |
| Hypothyroidism (%) | | 0.9 | 2.7 | < 0.001 |  |
| Liver disease (%) | | 1.8 | 1.1 | 0.036 |  |
| Lymphoma (%) | | 0.0 | 0.0 | 0.632 |  |
| Fluid and electrolyte disorders (%) | | 47.7 | 23.5 | < 0.001 |  |
| Metastatic cancer (%) | | 0.0 | 0.1 | 0.449 |  |
| Obesity (%) | | 0.5 | 0.8 | 0.226 |  |
| Paralysis (%) | | 6.4 | 0.8 | < 0.001 |  |
| Peripheral vascular disorders (%) | | 10.1 | 3.4 | < 0.001 |  |
| Psychoses (%) | | 0.5 | 0.1 | 0.001 |  |
| Pulmonary circulation disorders (%) | | 9.2 | 3.7 | < 0.001 |  |
| Renal failure (%) | | 3.7 | 0.6 | < 0.001 |  |
| Valvular disease (%) | | 30.3 | 20.8 | < 0.001 |  |
| Weight loss (%) | | 17.0 | 5.4 | <0.001 |  |

| **sTable 6. Univariate Analysis of Patient Characteristics and Postoperative Delirium Incidence in Large Small Bed-Size Hospitals** | | | | |  |
| --- | --- | --- | --- | --- | --- |
| Characteristics | | POD | No POD | P |  |
| Total (n=count) | | 476 | 23250 |  |  |
| Total incidence (%) | | 1.8 | |  |  |
| Age group (%) | |  |  |  |  |
|  | 0-2 | 70.4 | 70.8 | 0.036 |  |
|  | 3-5 | 8.4 | 9.8 |  |  |
|  | 6-12 | 16.0 | 12.2 |  |  |
|  | 13-17 | 5.3 | 7.2 |  |  |
| Gender (%) | |  |  |  |  |
|  | Male | 52.3 | 53.8 | 0.514 |  |
|  | Female | 47.7 | 46.2 |  |  |
| Race (%) | |  |  |  |  |
|  | White | 38.9 | 32.4 | < 0.001 |  |
|  | Black | 10.5 | 11.3 |  |  |
|  | Hispanic | 16.0 | 12.7 |  |  |
|  | Asian or Pacific Islander | 1.1 | 2.6 |  |  |
|  | Native American | 0.0 | 0.4 |  |  |
|  | Other | 33.6 | 40.6 |  |  |
| Type of insure (%) | |  |  |  |  |
|  | Medicare and Medicaid | 63.7 | 49.8 | < 0.001 |  |
|  | Private insurance | 30.5 | 41.7 |  |  |
|  | Self-pay | 0.0 | 1.3 |  |  |
|  | No charge | 1.7 | 1.0 |  |  |
|  | Other | 4.2 | 6.2 |  |  |
| Elective admission (%) | | 42.9 | 67.2 | < 0.001 |  |
| Type of hospital (teaching %) | | 100.0 | 99.0 | 0.055 |  |
| Location of hospital (urban, %) | | 100.0 | 100.0 |  |  |
| Region of hospital (%) | |  |  |  |  |
| Northeast | | 1.1 | 4.8 |  |  |
| Midwest or North Central | | 63.0 | 41.2 | < 0.001 |  |
| South | | 32.8 | 47.1 |  |  |
| West | | 3.2 | 7.0 |  |  |
| Died during hospitalization (%) | | 14.7 | 2.3 | < 0.001 |  |
| Deficiency anemia (%) | | 9.5 | 3.3 | < 0.001 |  |
| Chronic blood loss anemia (%) | | 2.1 | 0.5 | < 0.001 |  |
| Congestive heart failure (%) | | 27.3 | 13.6 | < 0.001 |  |
| Chronic pulmonary disease (%) | | 7.4 | 4.8 | 0.010 |  |
| Coagulopathy (%) | | 27.1 | 7.7 | < 0.001 |  |
| Depression (%) | | 2.1 | 0.4 | < 0.001 |  |
| Diabetes, uncomplicated (%) | | 0.0 | 0.0 | 0.650 |  |
| Diabetes with chronic complications (%) | | 1.1 | 0.0 | < 0.001 |  |
| Hypertension (%) | | 16.6 | 11.3 | < 0.001 |  |
| Hypothyroidism (%) | | 4.2 | 2.3 | 0.007 |  |
| Liver disease (%) | | 6.3 | 1.2 | < 0.001 |  |
| Lymphoma (%) | | 0.0 | 0.0 | 0.749 |  |
| Fluid and electrolyte disorders (%) | | 27.8 | 60.5 | < 0.001 |  |
| Metastatic cancer (%) | | 1.1 | 0.1 | < 0.001 |  |
| Obesity (%) | | 0.9 | 1.1 | 0.641 |  |
| Paralysis (%) | | 2.1 | 0.5 | < 0.001 |  |
| Peripheral vascular disorders (%) | | 13.7 | 3.8 | < 0.001 |  |
| Psychoses (%) | | 1.1 | 0.1 | < 0.001 |  |
| Pulmonary circulation disorders (%) | | 18.9 | 6.7 | < 0.001 |  |
| Renal failure (%) | | 3.2 | 0.5 | < 0.001 |  |
| Valvular disease (%) | | 35.7 | 27.8 | < 0.001 |  |
| Weight loss (%) | | 8.4 | 5.5 | 0.006 |  |

| **sTable 7. Univariate Analysis of Patient Characteristics and Postoperative Delirium Incidence in the Northeast Region** | | | | |
| --- | --- | --- | --- | --- |
| Characteristics | | POD | No POD | P |
| Total (n=count) | | 369 | 30413 |  |
| Total incidence (%) | | 1.2 | |  |
| Age (median, years) | | 0.0 (0.0 ,5.0) | 0.0 (0.0 ,3.0) | < 0.001 |
| Age group (%) | |  |  |  |
|  | 0-2 | 68.8 | 69.1 | < 0.001 |
|  | 3-5 | 6.8 | 11.7 |  |
|  | 6-12 | 9.5 | 11.5 |  |
|  | 13-17 | 14.9 | 7.7 |  |
| Gender (%) | |  |  |  |
|  | Male | 59.5 | 56.0 | 0.188 |
|  | Female | 40.5 | 44.0 |  |
| Race (%) | |  |  |  |
|  | White | 44.3 | 43.3 | < 0.001 |
|  | Black | 16.3 | 10.0 |  |
|  | Hispanic | 12.2 | 12.8 |  |
|  | Asian or Pacific Islander | 4.1 | 4.5 |  |
|  | Native American | 1.4 | 0.3 |  |
|  | Other | 21.7 | 29.2 |  |
| Type of insure (%) | |  |  |  |
|  | Medicare and Medicaid | 42.0 | 38.3 | 0.079 |
|  | Private insurance | 47.2 | 53.6 |  |
|  | Self-pay | 4.1 | 3.3 |  |
|  | No charge | 0.0 | 0.1 |  |
|  | Other | 6.8 | 4.8 |  |
| Bed size of hospital (%) | |  |  |  |
| Small | | 1.4 | 3.6 | 0.003 |
| Medium | | 40.7 | 33.6 |  |
| Large | | 58.0 | 62.7 |  |
| Elective admission (%) | | 43.6 | 62.5 | < 0.001 |
| Type of hospital (teaching %) | | 100.0 | 99.3 | 0.180 |
| Preoperative comorbidities | |  |  |  |
| Location of hospital (urban, %) | | 100.0 | 99.4 | 0.229 |
| Died during hospitalization (%) | | 9.5 | 2.9 | < 0.001 |
| Preoperative comorbidities | |  |  |  |
| Deficiency anemia (%) | | 1.4 | 3.3 | 0.050 |
| Chronic blood loss anemia (%) | | 1.4 | 0.1 | < 0.001 |
| Congestive heart failure (%) | | 20.3 | 8.9 | < 0.001 |
| Chronic pulmonary disease (%) | | 5.4 | 4.4 | 0.332 |
| Coagulopathy (%) | | 13.5 | 5.5 | < 0.001 |
| Depression (%) | | 0.0 | 0.2 | 0.360 |
| Diabetes, uncomplicated (%) | | 0.0 | 0.1 | 0.622 |
| Diabetes with chronic complications (%) | | 0.0 | 0.0 | 0.805 |
| Hypertension (%) | | 10.8 | 8.5 | 0.119 |
| Hypothyroidism (%) | | 4.1 | 2.9 | 0.173 |
| Liver disease (%) | | 4.1 | 0.7 | < 0.001 |
| Lymphoma (%) | | 100% | 100% | - |
| Fluid and electrolyte disorders (%) | | 43.1 | 23.8 | < 0.001 |
| Metastatic cancer (%) | | 1.4 | 0.2 | < 0.001 |
| Obesity (%) | | 0.0 | 0.6 | 0.144 |
| Paralysis (%) | | 2.7 | 0.6 | < 0.001 |
| Peripheral vascular disorders (%) | | 12.2 | 2.9 | < 0.001 |
| Psychoses (%) | | 0.0 | 0.1 | 0.582 |
| Pulmonary circulation disorders (%) | | 5.4 | 4.0 | 0.161 |
| Renal failure (%) | | 0.0 | 0.4 | 0.237 |
| Valvular disease (%) | | 19.0 | 20.5 | 0.473 |
| Weight loss (%) | | 14.9 | 5.2 | < 0.001 |

| **sTable 8. Univariate Analysis of Patient Characteristics and Postoperative Delirium Incidence in the Midwest/North Central Region** | | | | |
| --- | --- | --- | --- | --- |
| Characteristics | | POD | No POD | P |
| Total (n=count) | | 1008 | 37576 |  |
| Total incidence (%) | | 2.6 | |  |
| Age group (%) | |  |  |  |
|  | 0-2 | 73.7 | 70.0 | 0.003 |
|  | 3-5 | 8.9 | 11.1 |  |
|  | 6-12 | 7.9 | 10.6 |  |
|  | 13-17 | 9.4 | 8.4 |  |
| Gender (%) | |  |  |  |
|  | Male | 56.4 | 54.4 | 0.206 |
|  | Female | 43.6 | 45.6 |  |
| Race (%) | |  |  |  |
|  | White | 54.5 | 55.3 | 0.003 |
|  | Black | 12.8 | 10.8 |  |
|  | Hispanic | 5.0 | 6.8 |  |
|  | Asian or Pacific Islander | 3.0 | 2.5 |  |
|  | Native American | 1.0 | 0.4 |  |
|  | Other | 23.8 | 24.2 |  |
| Type of insure (%) | |  |  |  |
|  | Medicare and Medicaid | 49.0 | 45.5 | 0.164 |
|  | Private insurance | 46.5 | 49.5 |  |
|  | Self-pay | 1.0 | 1.5 |  |
|  | No charge | 0.0 | 0.0 |  |
|  | Other | 3.5 | 3.5 |  |
| Bed size of hospital (%) | |  |  |  |
|  | Small | 29.7 | 25.5 | < 0.001 |
|  | Medium | 15.9 | 21.0 |  |
|  | Large | 54.4 | 53.5 |  |
| Elective admission (%) | | 43.1 | 62.1 | < 0.001 |
| Type of hospital (teaching %) | | 98.5 | 99.4 | < 0.001 |
| Location of hospital (urban, %) | | 99.5 | 99.7 | 0.211 |
| Died during hospitalization (%) | | 18.3 | 3.4 | < 0.001 |
| Preoperative comorbidities | |  |  |  |
| Deficiency anemia (%) | | 9.9 | 3.8 | < 0.001 |
| Chronic blood loss anemia (%) | | 0.5 | 0.6 | 0.713 |
| Congestive heart failure (%) | | 29.8 | 10.4 | < 0.001 |
| Chronic pulmonary disease (%) | | 6.4 | 4.2 | 0.001 |
| Coagulopathy (%) | | 26.7 | 7.2 | < 0.001 |
| Depression (%) | | 1.0 | 0.5 | 0.026 |
| Diabetes, uncomplicated (%) | | 0.0 | 0.1 | 0.272 |
| Diabetes with chronic complications (%) | | 0.5 | 0.1 | < 0.001 |
| Hypertension (%) | | 14.9 | 10.0 | < 0.001 |
| Hypothyroidism (%) | | 3.5 | 3.3 | 0.796 |
| Liver disease (%) | | 4.0 | 1.1 | < 0.001 |
| Lymphoma (%) | | 0.0 | 0.0 | 0.714 |
| Fluid and electrolyte disorders (%) | | 59.4 | 27.7 | < 0.001 |
| Metastatic cancer (%) | | 0.5 | 0.1 | < 0.001 |
| Obesity (%) | | 0.5 | 0.8 | 0.306 |
| Paralysis (%) | | 5.5 | 0.8 | < 0.001 |
| Peripheral vascular disorders (%) | | 10.9 | 4.2 | < 0.001 |
| Psychoses (%) | | 1.0 | 0.1 | < 0.001 |
| Pulmonary circulation disorders (%) | | 16.9 | 5.8 | < 0.001 |
| Renal failure (%) | | 1.5 | 0.5 | < 0.001 |
| Valvular disease (%) | | 37.1 | 25.1 | < 0.001 |
| Weight loss (%) | | 11.4 | 4.5 | < 0.001 |

| **sTable 9. Univariate Analysis of Patient Characteristics and Postoperative Delirium Incidence in the South Region** | | | | |
| --- | --- | --- | --- | --- |
| Characteristics | | POD | No POD | P |
| Total (n=count) | | 1480 | 174207 |  |
| Total incidence (%) | | 2.0 | |  |
| Age group (%) | |  |  |  |
|  | 0-2 | 61.8 | 73.0 | < 0.001 |
|  | 3-5 | 6.1 | 7.5 |  |
|  | 6-12 | 24.8 | 13.1 |  |
|  | 13-17 | 7.4 | 6.4 |  |
| Gender (%) | |  |  |  |
|  | Male | 63.2 | 54.6 | < 0.001 |
|  | Female | 36.8 | 45.4 |  |
| Race (%) | |  |  |  |
|  | White | 44.4 | 43.8 | < 0.001 |
|  | Black | 16.1 | 17.8 |  |
|  | Hispanic | 24.6 | 20.3 |  |
|  | Asian or Pacific Islander | 1.8 | 2.4 |  |
|  | Native American | 1.0 | 0.5 |  |
|  | Other | 12.1 | 15.2 |  |
| Type of insure (%) | |  |  |  |
|  | Medicare and Medicaid | 66.2 | 56.6 | < 0.001 |
|  | Private insurance | 27.2 | 37.0 |  |
|  | Self-pay | 1.3 | 1.7 |  |
|  | No charge | 0.5 | 0.3 |  |
|  | Other | 4.7 | 4.4 |  |
| Bed size of hospital (%) | |  |  |  |
|  | Small | 10.5 | 14.8 | < 0.001 |
|  | Medium | 29.6 | 25.5 |  |
|  | Large | 59.9 | 59.8 |  |
| Elective admission (%) | | 42.2 | 60.5 | < 0.001 |
| Type of hospital (teaching %) | | 100.0 | 99.6 | 0.012 |
| Preoperative comorbidities | |  |  |  |
| Location of hospital (urban, %) | | 100.0 | 100.0 | 0.655 |
| Died during hospitalization (%) | | 10.7 | 3.0 | < 0.001 |
| Preoperative comorbidities | | | | |
| Deficiency anemia (%) | | 6.8 | 3.7 | < 0.001 |
| Chronic blood loss anemia (%) | | 1.0 | 0.3 | < 0.001 |
| Congestive heart failure (%) | | 19.2 | 11.9 | < 0.001 |
| Chronic pulmonary disease (%) | | 6.3 | 3.9 | < 0.001 |
| Coagulopathy (%) | | 23.2 | 7.7 | < 0.001 |
| Depression (%) | | 1.0 | 0.3 | < 0.001 |
| Diabetes, uncomplicated (%) | | 0.0 | 0.1 | 0.218 |
| Diabetes with chronic complications (%) | | 0.0 | 0.1 | 0.371 |
| Hypertension (%) | | 18.2 | 10.6 | < 0.001 |
| Hypothyroidism (%) | | 1.7 | 2.2 | 0.221 |
| Liver disease (%) | | 1.4 | 0.9 | 0.045 |
| Lymphoma (%) | | 0.0 | 0.0 | 0.655 |
| Fluid and electrolyte disorders (%) | | 51.3 | 27.3 | < 0.001 |
| Metastatic cancer (%) | | 0.0 | 0.0 | 0.439 |
| Obesity (%) | | 1.3 | 0.8 | 0.039 |
| Paralysis (%) | | 5.7 | 0.8 | < 0.001 |
| Peripheral vascular disorders (%) | | 7.8 | 3.1 | < 0.001 |
| Psychoses (%) | | 1.1 | 0.1 | < 0.001 |
| Pulmonary circulation disorders (%) | | 9.8 | 3.8 | < 0.001 |
| Renal failure (%) | | 3.7 | 0.4 | < 0.001 |
| Valvular disease (%) | | 26.3 | 21.1 | < 0.001 |
| Weight loss (%) | | 9.8 | 4.2 | <0.001 |

| **sTable10. Univariate Analysis of Patient Characteristics and Postoperative Delirium Incidence in the West Region** | | | | |
| --- | --- | --- | --- | --- |
| Characteristics | | POD | No POD | P |
| Total (n=count) | | 998 | 41220 |  |
| Total incidence (%) | | 2.4 | |  |
| Age group (%) | |  |  |  |
|  | 0-2 | 73.3 | 69.7 | 0.069 |
|  | 3-5 | 10.0 | 10.9 |  |
|  | 6-12 | 9.1 | 11.3 |  |
|  | 13-17 | 7.5 | 8.1 |  |
| Gender (%) | |  |  |  |
|  | Male | 57.3 | 54.8 | 0.108 |
|  | Female | 42.7 | 45.2 |  |
| Race (%) | |  |  |  |
|  | White | 43.7 | 40.4 | 0.005 |
|  | Black | 6.0 | 5..0 |  |
|  | Hispanic | 33.3 | 33.0 |  |
|  | Asian or Pacific Islander | 7.0 | 7.8 |  |
|  | Native American | 1.5 | 1.5 |  |
|  | Other | 8.5 | 12.2 |  |
| Type of insure (%) | |  |  |  |
|  | Medicare and Medicaid | 44.8 | 45.3 | 0.008 |
|  | Private insurance | 40.6 | 41.7 |  |
|  | Self-pay | 0.5 | 0.6 |  |
|  | No charge | 1.5 | 0.6 |  |
|  | Other | 12.5 | 11.7 |  |
| Bed size of hospital (%) | |  |  |  |
|  | Small | 1.5 | 3.9 | < 0.001 |
|  | Medium | 34.2 | 25.7 |  |
|  | Large | 64.3 | 70.4 |  |
| Elective admission (%) | | 48.2 | 63.1 | < 0.001 |
| Type of hospital (teaching %) | | 99.5 | 97.3 | < 0.001 |
|  | |  |  |  |
| Location of hospital (urban, %) | | 100.0 | 100.0 | - |
| Died during hospitalization (%) | | 12.6 | 3.2 | < 0.001 |
| Preoperative comorbidities | | | | |
| Deficiency anemia (%) | | 7.1 | 3.8 | < 0.001 |
| Chronic blood loss anemia (%) | | 0.0 | 0.4 | 0.034 |
| Congestive heart failure (%) | | 27.1 | 12.4 | < 0.001 |
| Chronic pulmonary disease (%) | | 3.6 | 3.6 | 0.972 |
| Coagulopathy (%) | | 22.6 | 7.0 | < 0.001 |
| Depression (%) | | 1.0 | 0.4 | 0.001 |
| Diabetes, uncomplicated (%) | | 0.0 | 0.1 | 0.394 |
| Diabetes with chronic complications (%) | | 0.0 | 0.0 | 0.486 |
| Hypertension (%) | | 11.1 | 9.7 | 0.147 |
| Hypothyroidism (%) | | 0.5 | 1.9 | 0.001 |
| Liver disease (%) | | 3.5 | 1.2 | < 0.001 |
| Lymphoma (%) | | 0.5 | 0.1 | < 0.001 |
| Fluid and electrolyte disorders (%) | | 57.8 | 23.7 | < 0.001 |
| Metastatic cancer (%) | | 0.0 | 0.1 | 0.436 |
| Obesity (%) | | 0.5 | 0.9 | 0.165 |
| Paralysis (%) | | 4.0 | 0.9 | < 0.001 |
| Peripheral vascular disorders (%) | | 4.5 | 3.6 | 0.121 |
| Psychoses (%) | | 0.0 | 0.1 | 0.357 |
| Pulmonary circulation disorders (%) | | 10.6 | 5.5 | < 0.001 |
| Renal failure (%) | | 3.1 | 0.5 | < 0.001 |
| Valvular disease (%) | | 25.1 | 22.7 | 0.072 |
| Weight loss (%) | | 13.0 | 4.3 | < 0.001 |

sTable 11. ****Variance inflation factors (VIFs) for all predictors in the final multivariable model****

| Characteristics | VIF | |
| --- | --- | --- |
| Age group | 1.188 | |
| Indicator of sex | 1.006 | |
| Race (uniform) |  | 1.0 |
| PAY1_new |  | 1.009 |
| Bed size of hospital (STRATA) |  | 1.042 |
| Elective versus non-elective admission |  | 1.116 |
| Teaching hospital |  | 1.209 |
| Urban hospital |  | 1.208 |
| Region of hospital (STRATA) |  | 1.021 |
| AHRQ comorbidity measure: Deficiency anemias |  | 1.021 |
| AHRQ comorbidity measure: Chronic blood loss anemia |  | 1.004 |
| AHRQ comorbidity measure: Congestive heart failure |  | 1.142 |
| AHRQ comorbidity measure: Chronic pulmonary disease |  | 1.040 |
| AHRQ comorbidity measure: Coagulopathy |  | 1.048 |
| AHRQ comorbidity measure: Depression |  | 1.033 |
| AHRQ comorbidity measure: Diabetes with chronic complications |  | 1.014 |
| AHRQ comorbidity measure: Hypertension (combine uncomplicated and complicated) |  | 1.024 |
| AHRQ comorbidity measure: Hypothyroidism |  | 1.007 |
| AHRQ comorbidity measure: Liver disease |  | 1.020 |
| AHRQ comorbidity measure: Lymphoma |  | 1.006 |
| AHRQ comorbidity measure: Fluid and electrolyte disorders |  | 1.061 |
| AHRQ comorbidity measure: Metastatic cancer |  | 1.008 |
| AHRQ comorbidity measure: Obesity |  | 1.043 |
| AHRQ comorbidity measure: Paralysis |  | 1.006 |
| AHRQ comorbidity measure: Peripheral vascular disorders |  | 1.020 |
| AHRQ comorbidity measure: Psychoses |  | 1.007 |
| AHRQ comorbidity measure: Pulmonary circulation disorders |  | 1.073 |
| AHRQ comorbidity measure: Renal failure |  | 1.011 |
| AHRQ comorbidity measure: Valvular disease | 1.080 | |
| AHRQ comorbidity measure: Weight loss |  | 1.055 |

| sTable 12. Absolute risk differences (ARDs) for predictors in the final multivariable model (ARDs to be calculated) | | |
| --- | --- | --- |
| Variable | OR (95% CI) | ARD (%) |
| Age group (vs. 0‑2 years) |  |  |
| 3‑5 years | 1.231 (1.087-1.395) |  |
| 6‑12 years | 1.827 (1.659-2.012) |  |
| 13‑17 years | 1.565 (1.391-1.761) |  |
| Female (vs. male) | 0.855 (0.733-0.996) | -0.4 |
| Race (vs. White) |  |  |
| Black | 0.970 (0.874-1.077) |  |
| Hispanic | 0.976 (0.892-1.067) |  |
| Asian or Pacific Islander | 0.919 (0.771-1.095) |  |
| Native American | 1.497 (1.103-2.033) |  |
| Other | 0.813 (0.738-0.895) |  |
| Insurance (vs. Medicare/Medicaid) |  |  |
| Private insurance | 0.803 (0.748-0.863) |  |
| Self‑pay | 0.740 (0.558-0.983) |  |
| No charge | 2.131 (1.391-3.262) |  |
| Other | 0.987 (0.863-1.129) |  |
| Bed size (vs. small) |  |  |
| Medium | 1.122 (1.001-1.257) |  |
| Large | 0.989 (0.890-1.090) |  |
| Elective admission (vs. non‑elective) | 0.431 (0.402-0.462) | -1.5 |
| Teaching hospital (vs. non‑teaching) | 2.584 (1.549-4.311) | 1.0 |
| Urban hospital (vs. rural) | 0.352 (0.126-0.980) | 0.5 |
| Region (vs. Northeast) |  |  |
| Midwest or North Central | 2.171 (1.920-2.454) |  |
| South | 1.520 (1.353-1.709) |  |
| West | 1.923 (1.699-2.176) |  |
| Deficiency anemia | 1.492 (1.309-1.699) | 1.9 |
| Chronic blood loss anemia | 1.045 (0.696-1.570) | 1.5 |
| Congestive heart failure | 1.626 (1.493-1.772) | 2.5 |
| Chronic pulmonary disease | 1.287 (1.115-1.486) | 0.9 |
| Coagulopathy | 2.419 (2.228-2.626) | 4.7 |
| Depression | 1.752 (1.219-2.518) | 3.5 |
| Diabetes with chronic complications | 0.920 (0.360-2.355) | 2.9 |
| Hypertension | 1.188 (1.083-1.306) | 1.0 |
| Hypothyroidism | 0.607 (0.483-0.762) | -0.4 |
| Liver disease | 1.445 (1.174-1.779) | 3.9 |
| Lymphoma | 3.350 (1.248-8.994) | 9.0 |
| Fluid and electrolyte disorders | 2.570 (2.402-2.749) | 1.9 |
| Metastatic cancer | 2.623 (1.332-5.167) | 4.7 |
| Obesity | 0.691 (0.471-1.013) | 0.9 |
| Paralysis | 4.686 (3.980-5.519) | 9.8 |
| Peripheral vascular disorders | 1.880 (1.663-2.125) | 2.8 |
| Psychoses | 4.656 (2.998-7.232) | 9.2 |
| Pulmonary circulation disorders | 1.490 (1.334-1.664) | 3.0 |
| Renal failure | 2.964 (2.372-3.703) | 8.6 |
| Valvular disease | 1.025 (0.949-1.107) | 0.7 |
| Weight loss | 1.643 (1.474-1.831) | 3.3 |
| Acute renal failure | 3.325 (3.076-3.595) | 6.1 |
| Acute myocardial infarction | 1.718 (1.528-1.931) | 2.7 |
| Pneumonia | 3.448 (3.138-3.790) | 7.8 |
| Blood transfusion | 0.867 (0.806-0.932) | -0.1 |
| Continuous mechanical ventilation | 1.801 (1.676-1.936) | 1.7 |
| Urinary tract infection | 1.601 (1.394-1.838) | 4.4 |
| Wound dehiscence | 1.553 (1.320-1.826) | 2.8 |
| arrhythmia | 1.292 (1.142-1.461) | 1.2 |
| Postoperative shock | 1.914 (1.619-2.263) | 5.1 |
| respiratory failure | 2.024 (1.881-2.177) | 3.1 |
